# Network analysis of noncoding RNAs in pepper provides insights into fruit ripening control

**DOI:** 10.1038/s41598-019-45427-1

**Published:** 2019-06-19

**Authors:** Jinhua Zuo, Yunxiang Wang, Benzhong Zhu, Yunbo Luo, Qing Wang, Lipu Gao

**Affiliations:** 1 Key Laboratory of Vegetable Postharvest Processing, Ministry of Agriculture, Beijing Key Laboratory of Fruits and Vegetable Storage and Processing, Key Laboratory of Biology and Genetic Improvement of Horticultural Crops (North China) of Ministry of Agriculture, Key Laboratory of Urban Agriculture (North) of Ministry of Agriculture, Beijing, Vegetable Research Center, Beijing Academy of Agriculture and Forestry Sciences, Beijing, 100097 China; 2 Boyce Thompson Institute for Plant Research, Cornell University Campus, Ithaca, NY 14853 USA; 3 Beijing Academy of Forestry and Pomology Sciences, Beijing Academy of Agriculture and Forestry Sciences, Beijing, 100093 China; 4 Laboratory of Postharvest Molecular Biology of Fruits and Vegetables, Department of Food Biotechnology, College of Food Science and Nutritional Engineering, China Agricultural University, Beijing, 100083 China

**Keywords:** High-throughput screening, Next-generation sequencing

## Abstract

Pepper is an important vegetable worldwide and is a model plant for nonclimacteric fleshy fruit ripening. Drastic visual changes and internal biochemical alterations are involved in fruit coloration, flavor, texture, aroma, and palatability to animals during the pepper fruit ripening process. To explore the regulation of bell pepper fruit ripening by noncoding RNAs (ncRNAs), we examined their expression profiles; 43 microRNAs (miRNAs), 125 circular RNAs (circRNAs), 366 long noncoding RNAs (lncRNAs), and 3266 messenger RNAs (mRNAs) were differentially expressed (DE) in mature green and red ripe fruit. Gene Ontology (GO) and Kyoto Encyclopedia of Genes and Genomes (KEGG) analyses revealed that the targets of the DE ncRNAs and DE mRNAs included several kinds of transcription factors (TFs) (ERF, bHLH, WRKY, MYB, NAC, bZIP, and ARF), enzymes involved in cell wall metabolism (beta-galactosidase, beta-glucosidase, beta-amylase, chitinase, pectate lyase (PL), pectinesterase (PE) and polygalacturonase (PG)), enzymes involved in fruit color accumulation (bifunctional 15-cis-phytoene synthase, 9-cis-epoxycarotenoid dioxygenase, beta-carotene hydroxylase and carotene epsilon-monooxygenase), enzymes associated with fruit flavor and aroma (glutamate-1-semialdehyde 2,1-aminomutase, anthocyanin 5-aromatic acyltransferase, and eugenol synthase 1) and enzymes involved in the production of ethylene (ET) (ACO1/ACO4) as well as other plant hormones such as abscisic acid (ABA), auxin (IAA), and gibberellic acid (GA). Based on accumulation profiles, a network of ncRNAs and mRNAs associated with bell pepper fruit ripening was developed that provides a foundation for further developing a more refined understanding of the molecular biology of fruit ripening.

## Introduction

Pepper (*Capsicum annuum* L.) is currently the second most important vegetable worldwide, and the major pepper production areas are located in southern European countries^1^. Pepper is an example of a nonclimacteric fleshy fruit species, as it exhibits neither respiratory burst nor ethylene (ET) responses during the fruit ripening process^2,3^. Fruits of pepper are popular for their attractive color, pungency, distinct aroma and ability to be consumed at different ripening stages; moreover, pepper fruits are rich in various vitamins, carotenoids, and flavonoids and have potential health-promoting properties^3^. Pepper is also a model plant for fruit ripening research because its ripening process involves drastic visual changes and internal biochemical alterations involved in fruit coloration, flavor, texture, aroma, and palatability to animals^4–6^.

To date, thousands of noncoding RNAs (ncRNAs), which play important roles in different physiological and biochemical processes, have been identified in plants^7–9^. NcRNAs can be divided into different varieties, and the most common varieties are small ncRNAs (sRNAs), long ncRNAs (lncRNAs) and circular RNAs (circRNAs)^10^. Although numerous ncRNAs have been identified in several model plants, the functions of these ncRNAs are largely unknown. In the past few years, substantial progress has been made in deciphering the regulatory functions of sRNAs in the posttranscriptional regulation of gene expression^11,12^. LncRNAs can exercise their regulatory roles by sequence complementarity or homology with RNAs or DNA in either cis or trans configurations, regulating gene expression at the transcriptional or posttranscriptional level; moreover, lncRNAs can also regulate micro RNA (miRNA) activity as endogenous target mimics (eTMs) of miRNAs^13,14^. Thousands of circRNAs have recently been characterized, which furthers our understanding of RNA; however, although circRNAs reportedly play important roles in a range of biological processes in humans and animals^15,16^, knowledge of the functions of circRNAs in fruit plants is limited^9,17,18^.

Due to the rapid development of next-generation sequencing technology and bioinformatics, numerous ncRNAs have been identified in model plants^19–21^. To date, more than 300 conserved and novel miRNAs have been identified in different pepper varieties, and several miRNAs have been found to be involved in pepper fruit development and quality^22–25^. In addition, 6527 lncRNAs were found in hot pepper, and several of these lncRNAs were found during the fruit development process^26^. To better understand the functions of ncRNAs in bell pepper fruit ripening, deep sequencing and bioinformatic analysis were employed, leading to the identification of 43 differentially expressed (DE) miRNAs, 125 DE circRNAs, 366 DE lncRNAs, and 3266 DE mRNAs during pepper fruit ripening. In addition, the targets of the DE ncRNAs were also analyzed, and several targets were found to be involved in fruit color accumulation (such as beta-carotene hydroxylase 1/2, 15-cis-phytoene synthase, lycopene epsilon cyclase, and phytoene synthase 2), fruit flavor and aroma formation (such as glutamate-1-semialdehyde 2,1-aminomutase, alanine aminotransferase 2, and eugenol synthase 1), fruit texture (such as beta-galactosidase, cellulose synthase, and polygalacturonase (PG)) and ET and other hormone pathways (such as ACO1/4, ERF1, EIN3, ARF, gibberellic acid (GA), and auxin (IAA)). Furthermore, the networks of competing endogenous RNAs (ceRNAs) of miRNAs, circRNAs, lncRNAs and mRNAs were assessed using gene annotation to identify influenced pathways and processes.

## Materials and Methods

### Sample collection and preparation

Bell pepper fruit (*Capsicum annuum* L. cv. *Suoma*) in the mature green (40 d) and red ripe stages (60 d) was harvested after anthesis (d.p.a.) from a greenhouse in “Xiaotangshan” and quickly transported to the laboratory. Then, pericarp samples were collected from the bell pepper fruit (with 3 replicates per stage), frozen in liquid nitrogen and stored at −80 °C for the next experiment.

### Methods of RNA extraction and detection

The RNA samples were extracted with TRIzol. RNA quality was checked by assessing the RNA integrity number (RIN) with an Agilent 2100 Bioanalyzer (Agilent Technologies, CA, USA) and by measuring the RNA concentration with a NanoDrop 2000 Spectrophotometer (Thermo Fisher Scientific, Wilmington, DE) to ensure the use of qualified samples for sequencing. The library preparation for sRNA sequencing and the library preparation for lncRNA and circRNA sequencing were the same as previously reported^27^.

### Sequencing and ncRNA identification

The library preparations were sequenced on an Illumina Hiseq platform, and paired-end reads were generated. The raw data were filtered and all downstream analyses were based on clean data with high quality^27^. The known miRNAs and novel miRNAs were predicted using miRDeep2 software by comparison with known miRNAs from miRBase^28^. The CIRI (CircRNA Identifier) tools was used to identify circRNAs, scan SAM files twice and collect enough information to identify and characterize circRNAs, similar to previous reports^29^. The transcriptome was assembled using StringTie based on the reads mapped to the reference genome. The assembled transcripts were annotated using the gffcompare program, the different types of lncRNAs, including long intergenic ncRNAs (lincRNAs), intronic lncRNAs, antisense lncRNAs, and sense lncRNAs, were selected using cuffcompare previously reported^27^.

### Quantification of expression levels and DEG analysis of ncRNAs

MiRNA expression levels were estimated for each sample. sRNAs were mapped back onto the precursor sequence, and the read count for each miRNA was obtained from the mapping results. StringTie (1.3.1) was used to calculate the fragments per kilobase of transcript per million mapped reads (FPKMs) of both lncRNAs and coding genes in each sample, and the FPKM values of genes were computed by summing the FPKM values of transcripts in each gene group. The FPKM value was calculated based on the length of the fragments and the number of reads mapped to these fragments. The expression of circRNAs was determined by the number of junction reads identified by CIRI. Differential expression analysis of two conditions/groups was performed as previously reported^27^.

### GO and KEGG analyses and Network analysis of ceRNAs of ncRNA

Gene function was annotated as previously reported and Gene Ontology (GO) enrichment analysis of the differentially expressed genes (DEGs) was implemented by the GOseq R package-based Wallenius noncentral hypergeometric distribution, the KOBAS software was used to test the statistical enrichment of differentially expressed genes in KEGG pathways^27,30^. The ceRNAs network analysis of the ncRNAs were the same as previously reported^27,31^.

## Results

### Identification of DE and novel miRNAs, circRNAs, lncRNAs and mRNAs

**Figure 1:**
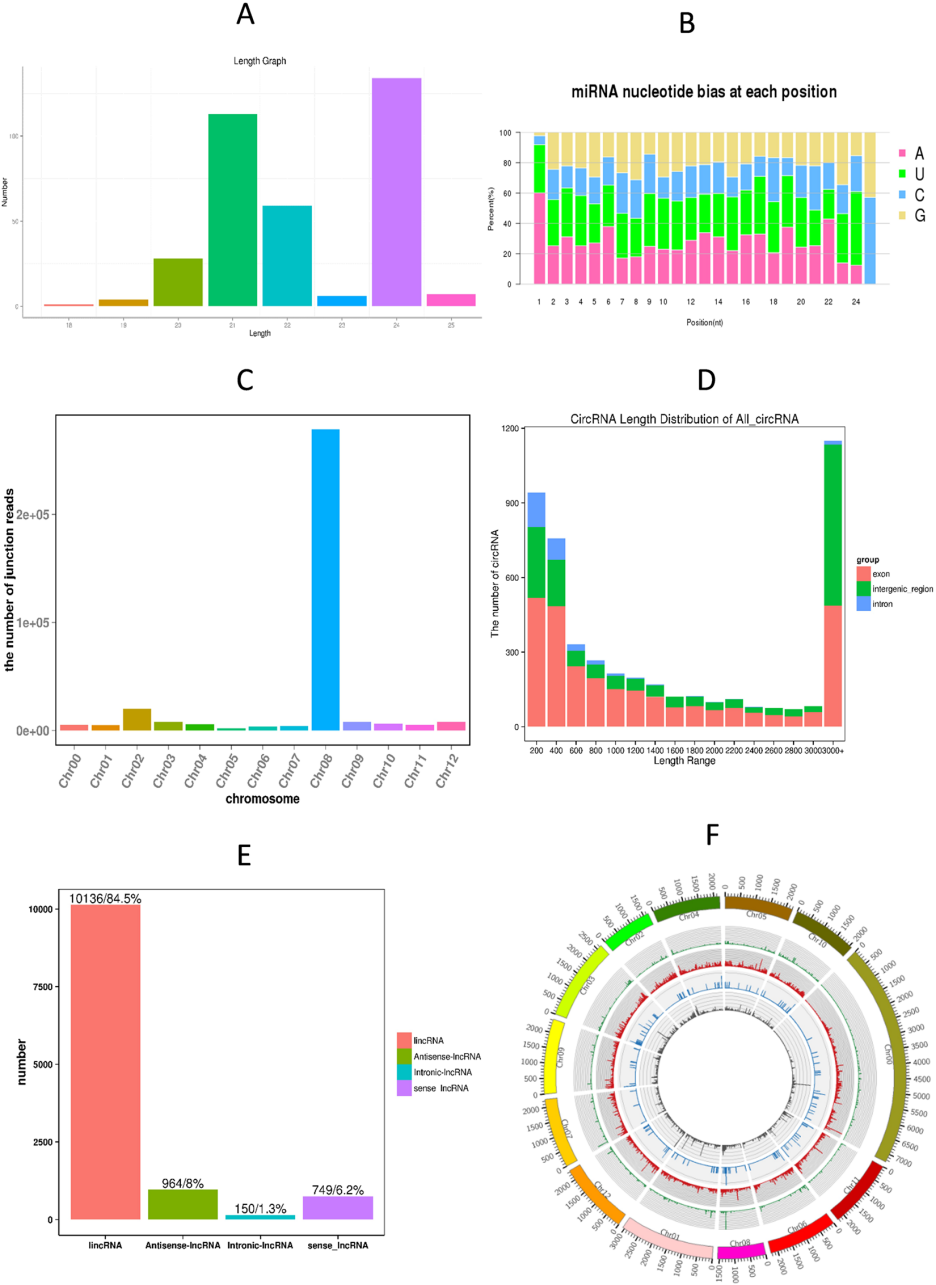
Information for the ncRNAs: All the miRNAs were mainly between 21 nt and 24 nt in length. The length of circRNAs was mainly between 200 nt and 400 nt. Most of the lncRNAs were lincRNAs, followed by antisense lncRNAs, sense lncRNAs and then intronic lncRNAs.

A total of 352 miRNAs were found in our libraries, of which 130 were known miRNAs and 222 were novel miRNAs. Most of the novel miRNAs were between 21 nt and 24 nt in length. We also analyzed the nucleotide bias for all the known and novel miRNAs; intriguingly, the most common nucleic acid bases were A and U, while the least common ones were G and C (Fig. 1A,B). The lengths of numerous circRNAs were between 200 nt and 400 nt, and a large number of the circRNAs were over 3000 nt in length. The majority of the circRNAs were from exons and intergenic regions. A total of 4795 novel circRNAs were found in our study, and most of them were on chromosome 8 (Fig. 1C,D). In addition, 11999 lncRNAs were found in bell pepper fruit, of which 194 were known lncRNAs and 11805 were novel lncRNAs. Interestingly, we found 139 lncRNAs that were the precursors of known and novel miRNAs, such as miR167, miR168, miR482, and miR5303 (Appendix 1). Among these lncRNAs, most were lincRNAs (10136, 84.5%), followed by antisense lncRNAs (964, 8%), sense lncRNAs (749, 6.2%) and then intronic lncRNAs (150, 1.3%) (Fig. 1E,F).

**Figure 2:**
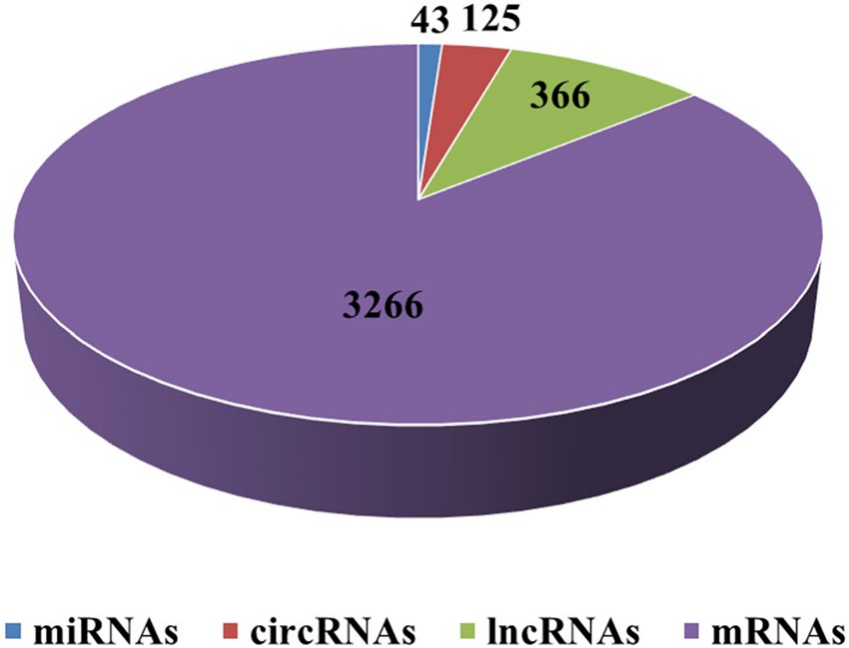
The distribution of DE ncRNAs in bell pepper fruit: 43 miRNAs, 125 circRNAs, 366 lncRNAs, and 3266 mRNAs were DE between the mature green and red ripe stages.

**Figure 3:**
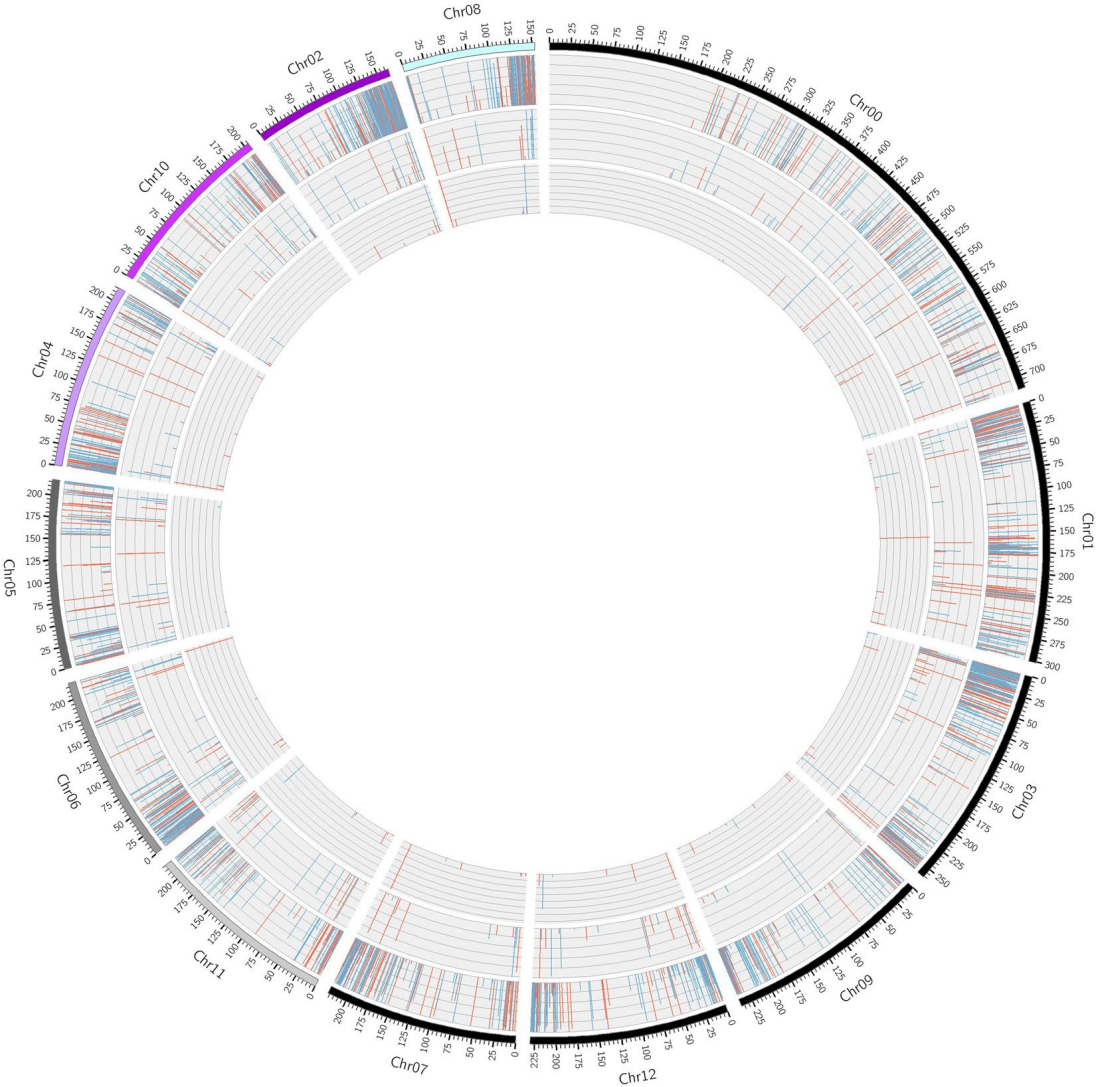
The distribution of the DE circRNAs, lncRNAs and mRNAs: the DE circRNAs were mostly distributed on chromosomes 1, 7, 8 and 9; the DE lncRNAs were mainly distributed on chromosomes 1, 3, 6 and 10; and the DE mRNAs were chiefly distributed on chromosomes 1, 2, 3, 6 and 8.

We compared the expression profiles of miRNAs, circRNAs, lncRNAs and mRNAs between mature green and red ripe bell pepper fruit and found that 43 miRNAs, 125 circRNAs, 366 lncRNAs, and 3266 mRNAs were DE (Fig. 2, Appendix 1). Among these DE RNAs, 32 miRNAs, 82 circRNAs, 186 lncRNAs, and 1188 mRNAs were upregulated, and 11 miRNAs, 43 circRNAs, 180 lncRNAs, and 2078 mRNAs were downregulated in the red ripe stage compared with the green ripe stage. The DE ncRNAs are listed in Appendix 1. The DE circRNAs were mostly found on chromosomes 1, 7, 8 and 9, and the number distributed on chromosome 7 was the largest. The DE lncRNAs were mainly distributed on chromosomes 1, 3, 6 and 10, while the DE mRNAs were chiefly distributed on chromosomes 1, 2, 3, 6 and 8; the number on chromosome 3 was the largest (Fig. 3).

### DE ncRNAs target parsing involved in pepper fruit ripening

**Figure 4:**
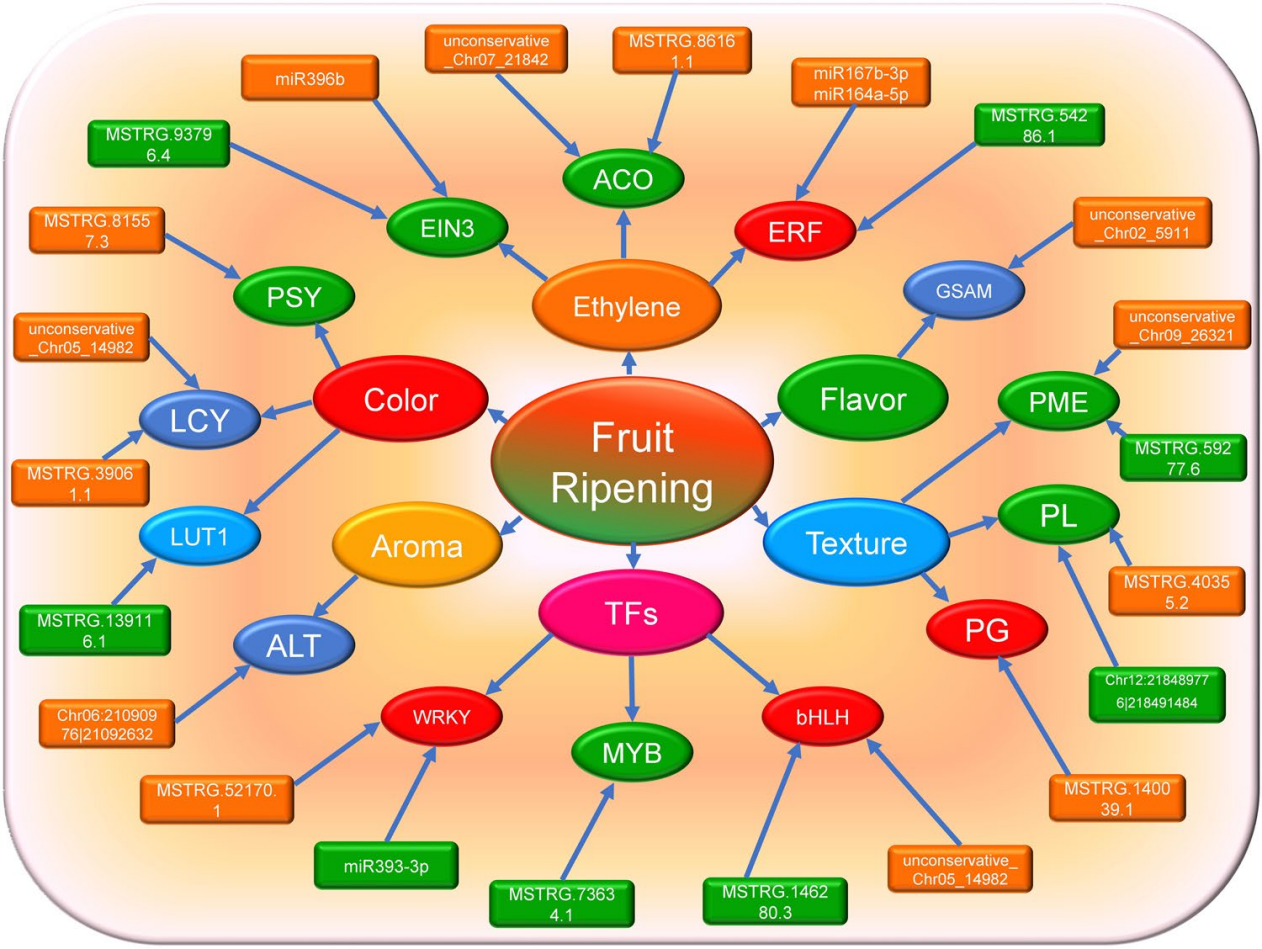
The ncRNA targets involved in fruit ripening: The targets of the DE ncRNAs were found to be associated with fruit color accumulation, fruit flavor and aroma formation, fruit texture, ET and several types of TFs.

The targets of the DE miRNAs were predicted, and a total of 909 targets were found (Appendix 2). The most enriched GO terms related to biological processes included single-organism cellular process, response to oxygen-containing compound, response to abiotic stimulus, response to organic substance, and single-organism transport. However, the most relevant GO terms associated with molecular functions were protein binding, sequence-specific DNA binding transcript, identical protein binding, protein serine/threonine kinase activity, phosphotransferase activity, and divalent inorganic cation transmembrane. KEGG pathway analysis suggested that mRNAs were remarkably enriched in the pathways involved in plant-pathogen interaction, RNA degradation, protein processing in the endoplasmic reticulum, plant hormone signal transduction, and RNA transport (Appendix 2). Intriguingly, several targets of the DE miRNAs were found to be involved in the fruit ripening process, such as ACO1, RTR2, EIN3, ERF1, ARF6/8, beta-glucosidase, pectinesterase (PE), lycopene epsilon cyclase, and squamosa promoter-binding-like protein (Fig. 4, Appendix 2).

To explore the potential function of DE circRNAs, GO and KEGG pathway analyses of circRNAs were also performed. The most relevant GO terms associated with biological processes were anatomical structure formation involved in morphogenesis, cytoskeleton-dependent cytokinesis, defense response to fungus, response to jasmonic acid, external encapsulating structure organization, and the L-glutamate biosynthetic process. The most relevant GO terms associated with molecular functions were phosphatidylinositol phosphate 5-phosphatase activity and 3 iron, 4 sulfur cluster binding. However, the KEGG pathways included only spliceosome and biosynthesis of amino acids (Appendix 4). In addition, several targets of the circRNAs were also found to be involved in the fruit ripening process, such as beta-galactosidase and pectate lyase (PL) (Fig. 4, Appendix 2).

To explore the potential functions of the DE ncRNAs, we performed GO and KEGG analyses. Both the cis and trans targets of the DE lncRNAs were analyzed, and 3266 targets were found. The relevant GO terms associated with biological processes and molecular functions included many important transcription factors (TFs) and key enzymes involved in pepper fruit ripening, such as MYB, NAC, WRKY, SBP, bHLH, bZIP, ACO1/4, MADS-box protein, EIN3, ERF, 15-cis-phytoene synthase, 9-cis-epoxycarotenoid dioxygenase NCED1, carotene epsilon-monooxygenase, beta-carotene hydroxylase, beta-galactosidase, PG, beta-amylase, beta-glucosidase, catalase chitinase, peroxidase, LOX, phenylalanine ammonia-lyase, abscisic acid (ABA), IAA, ARF, and GA (Fig. 4, Appendix 2). The KEGG analysis results showed that the most frequently predicted pathways were involved in carbon metabolism, ribosomes, plant hormone signal transduction, purine metabolism, biosynthesis of amino acids, and protein processing in the endoplasmic reticulum, indicating the specific regulatory functions of lncRNAs in fruit ripening in bell pepper (Fig. 4, Appendix 2).

### Comparative analysis of lncRNAs/mRNAs and functional annotation of DE mRNAs

**Figure 5:**
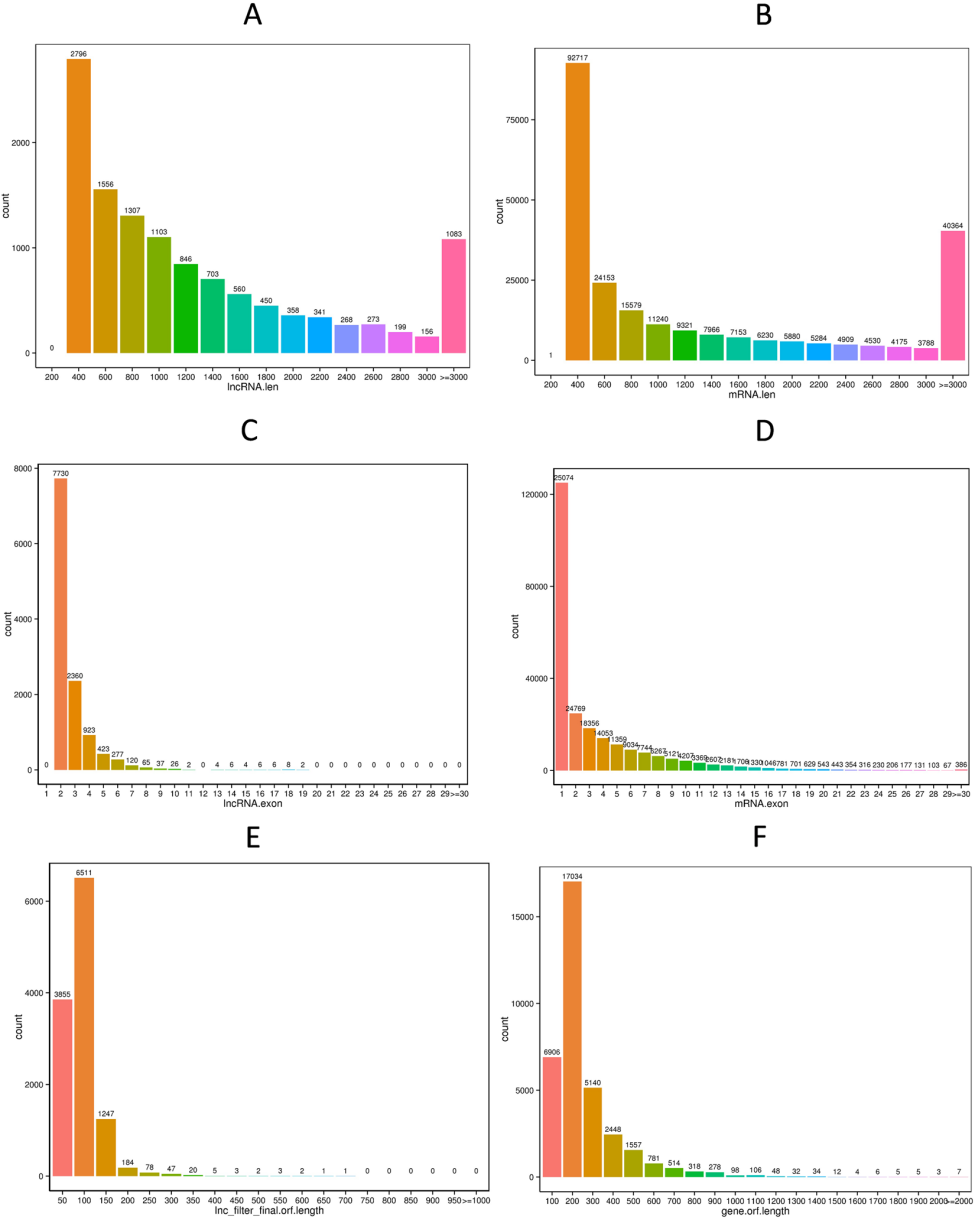
Comparison of lncRNAs and mRNAs: The length of lncRNAs and mRNAs, the number of exons of lncRNAs and mRNAs and the length of the open reading frames of lncRNAs and mRNAs were showed different distributions.

There are many differences between lncRNAs and mRNAs, including their length, exon numbers, and open reading frame. The isoforms and interactive expression of lncRNAs and mRNAs were also examined, and the distributions of these ncRNAs on the different chromosomes are described in Appendix 4. For lncRNAs with a length <3000 nt, the number declined with increasing length (Fig. 5A), and most lncRNAs had a length <1000 nt or ≥3000 nt. The length of mRNAs showed the same trends and exhibited two peaks at approximately 400 nt and ≥3000 nt (Fig. 5B). The corresponding number of lncRNAs distributed in the range of exons were smaller than the mRNAs and mainly below 10; however, the corresponding numberof mRNAs distributed in the range of exons were ranged from 1 to 30 (Fig. 5C,D). The length of corresponding open reading frames of lncRNAs was mainly between 50 nt and 150 nt (Fig. 5E), while the length of corresponding open reading frames of mRNAs was mainly between 100 nt and 900 nt (Fig. 5F).

**Figure 6:**
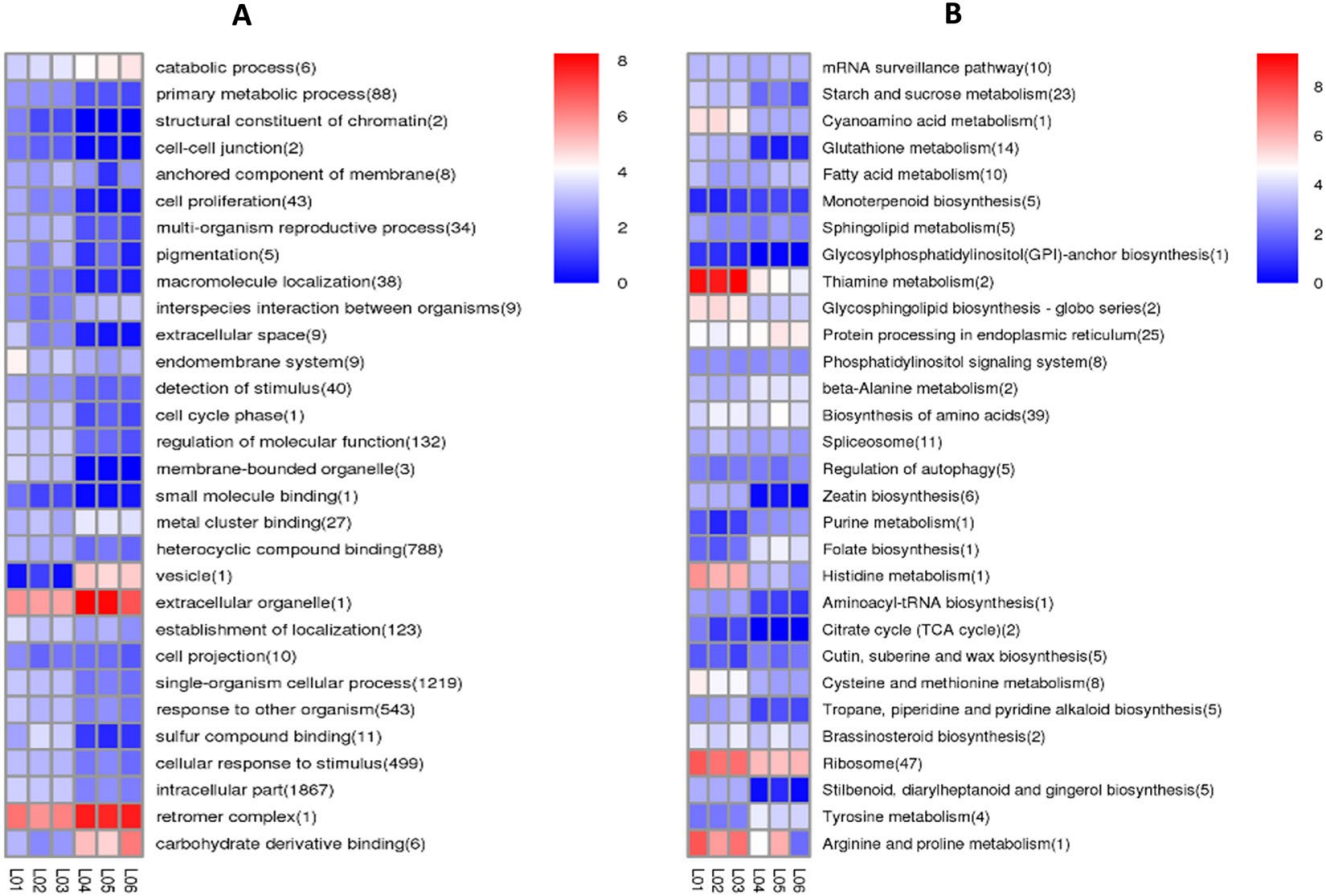
GO and KEGG clustering of DE mRNAs: The GO and KEGG cluster analyses revealed several DE mRNAs involved in different pathways, some of which were found to take part in the fruit ripening process.

For DE mRNAs, the most relevant GO terms associated with molecular functions were protein binding, sequence-specific DNA binding transcription factor activity, protein kinase activity, serine-type endopeptidase activity, protein serine/threonine kinase activity, transmembrane receptor protein kinase activity, flavonoid 3′-monooxygenase activity, and chlorophyll binding. KEGG pathway analysis indicated that the most frequently predicted pathways were involved in plant hormone signal transduction, carbon metabolism, ribosome, biosynthesis of amino acids, phenylpropanoid biosynthesis, starch and sucrose metabolism (Fig. 6). We found that many DE mRNAs were involved in pepper fruit ripening-related processes and could be divided into five different groups: the first group included enzymes associated with fruit texture such as PE, PL, beta-glucosidase, pectin acetylesterase, PG, and pectin methyltransferase; the second group included enzymes involved in fruit pigment accumulation such as 15-cis-phytoene desaturase, 9-cis-epoxycarotenoid dioxygenase NCED1, beta-carotene hydroxylase 1, phytoene synthase (PSY), lycopene epsilon cyclase, and carotenoid cleavage dioxygenase, and some of these enzymes were also found to be upregulated during chili pepper development^32^; the third group included components associated with fruit flavor or aroma such as 2-C-methyl-D-erythritol 2,4-cyclodiphosphate synthase, bifunctional phosphatase IMPL2, delta(7)-sterol-C5(6)-desaturase, and glutamate decarboxylase; and the fourth group included components associated with plant hormone-related processes such as ACO, MADS-box, EIN3, ERF, ABA, IAA, GA, and ARF. In addition, a fifth group included several TFs, such as MYB, bHLH, WRKY, and NAC (Appendix 4).

### Network analysis of the ncRNAs involved in fruit ripening

**Figure 7:**
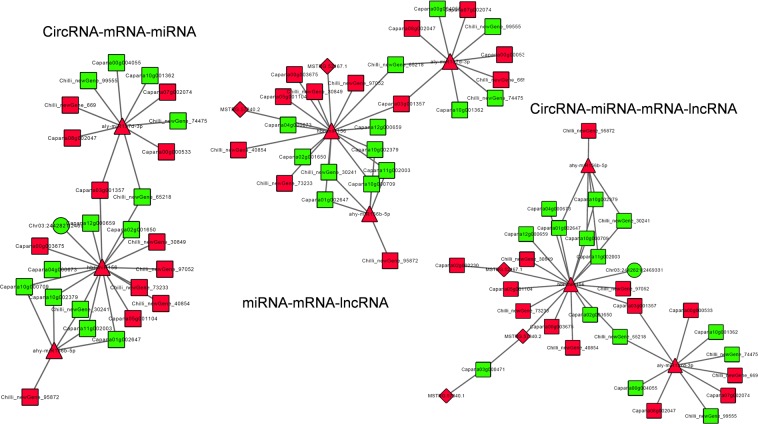
Network analysis of ncRNAs and mRNAs: The networks of three types including circRNA-mRNA-miRNA network, miRNA-mRNA-lncRNA and circRNA-miRNA-mRNA-lncRNA network were constructed.

As reported previously, circRNAs can act as “miRNA sponges”, and lncRNAs can be the precursors or targets of miRNAs. To explore coordinated regulatory functions, analysis of circRNA-mRNA-miRNA, miRNA-mRNA-lncRNA and circRNA-miRNA-mRNA-lncRNA networks was performed. In the circRNA-mRNA-miRNA network analysis, 9 circRNAs, 35 miRNAs and 84 mRNAs were found to be involved in the network; however, in the miRNA-mRNA-lncRNA network analysis, 34 miRNAs, 4 lncRNAs and 84 mRNAs were found in the network. Furthermore, in the circRNA-miRNA-mRNA-lncRNA network analysis, 9 circRNAs, 35 miRNAs, 124 lncRNAs and 256 mRNAs were involved in network regulation. Intriguingly, several biological processes were involved in the various fruit ripening processes, such as ethylene biosynthesis and signal transduction (ACO1, ACO4 and ERF039), fruit color formation (bifunctional 15-cis-phytoene synthase and lycopene epsilon cyclase), fruit texture (PG, pectin acetylesterase, and beta-galactosidase), and fruit flavor and aroma (alpha-farnesene synthase and anthocyanin 5-aromatic acyltransferase), indicating that their specific networks were involved in the coordinated regulation of the fruit ripening processes (Fig. 7, Appendix 5).

LncRNAs, circRNAs and mRNAs can interact with miRNAs through microRNA response elements (MREs) within ceRNA networks^9,33,34^. We developed candidate ceRNA relationships based on miRNA-target relationships and obtained 271 pairs of ceRNA relationships, including 148 pots. Then, we extracted three comprehensive ceRNA networks from the ceRNA relationship pairs, including 10 mRNAs and 20 circRNAs. More importantly, we found two important pathways, namely, the ERF TF, which plays important roles in ET signal transduction, and eugenol synthase 1, which may participate in pepper flavor formation, in the ceRNA regulatory network (Appendix 6).

## Discussion

Pepper is a typical nonclimacteric fruit and has emerged as a model for research on fruit ripening^35^. Fruit ripening is a highly coordinated biological process and involves numerous morphological and physiological processes, including pigment accumulation, nutritional changes, texture variation and metabolism of flavor and aromatic substances^36,37^. With the advancement of sequencing technology, increasing numbers of ncRNAs are being found in model fruit plants^9,38^. Several miRNAs and lncRNAs have been identified in different kinds of pepper varieties, but their functions are largely unknown. Here, in our research, we identified 222, 4795 and 11085 novel miRNAs, circRNAs and lncRNAs, respectively, which enrich the ncRNA database. Furthermore, 43 miRNAs, 125 circRNAs, 366 lncRNAs, and 3266 mRNAs were DE during fruit ripening, and several ncRNAs’ targets were found to be associated with fruit color, texture, flavor, aroma, hormones and several TFs, indicating that they have specific regulatory roles in the ripening of bell pepper fruit.

Color change is visible and is used as an indicator of fruit ripening; red peppers accumulate higher levels of total carotenoids during the ripening process^39,40^. The expression levels of some carotenoid biosynthetic genes encoding key carotenoid biosynthetic enzymes, such as PSY, phytoene desaturase (PDS), and capsanthin-capsorubin synthase (CCS), are higher in red pepper fruits than in pepper fruits of other colors; however, some of these genes are not expressed in peppers with lower levels of total carotenoids^39,41,42^. We found that one miRNA’s (unconservative_Chr05_14982) target is lycopene epsilon cyclase (Capana09g000177), which is a key enzyme in carotenoid synthesis and showed opposite expression patterns^43^. We also found that two lncRNAs (MSTRG.81557.3 and MSTRG.81557.4) target 15-cis-phytoene synthase, which is a key enzyme in carotenoid synthesis and showed the same expression^44^. Several lncRNAs (MSTRG.139116.1, MSTRG.139115.1, MSTRG.139111.1, and MSTRG.139117.1) target carotene epsilon-monooxygenase, which is a key enzyme in the lutein pathway^45^. In addition, we found that one miRNA (unconservative_Chr02_5911) targets glutamate-1-semialdehyde 2,1-aminomutase (Capana03g001893), which is an enzyme involved in tomato flavor synthesis^46^; one circRNA (Chr06:21090976|21092632) targets alanine aminotransferase 2 (Capana06g001160); and several lncRNAs target anthocyanin 5-aromatic acyltransferase (Capana10g000432), which contributes to pepper aroma formation^47^. One ncRNA can target several different genes, and different miRNAs, lncRNAs and circRNAs can also have the same target; however, the method through which they regulate their target genes synergistically to control pigment accumulation during pepper ripening is still largely unknown and needs further research.

Fruit ripening and softening involve numerous modifications to cell-wall polysaccharides, and during the bell pepper fruit ripening process, a dramatic change in the texture of the walls also occurs. Several enzymes, such as PG, pectin methyl esterase (PME), PL, and β-galactosidase, are involved in fruit ripening and softening^48^. We found that two known miRNAs (miR157d-3p and miR396h) target beta-glucosidase, which is an important enzyme in the fruit ripening process^49^ and that a novel miRNA (unconservative_Chr05_14982) targets cellulose synthase, which takes part in fruit cell wall formation^50^. Furthermore, several circRNAs and lncRNAs target beta-galactosidase, which plays important roles in the bell pepper ripening process^48^. In addition, several lncRNAs and circRNAs target PG, PL and PE, which are key enzymes in the fruit softening process^9^.

Fruit ripening is also affected by various hormones, such as ET, IAA, and ABA^35,36,51^. For example, IAA is known to retard tomato ripening by regulating ET and ABA^34^. We found that several DE ncRNA targets are important enzymes or TFs in the fruit ripening process, which is consistent with the results of previous studies^23,26^. Several lncRNAs and miRNAs target ACO1 and ACO4, which are important enzymes in ET biosynthesis, and several miRNAs (miR164a-5p, miR167b-3p, miR168a-3p, miR396b, miR482a and unconservative_Chr06_17217) target ETR2, EIN3 and ERFs, which are important regulators in the ET signal transduction pathway^36,52,53^. Previous studies reported that ARFs were targets of the can-miR160 and can-miR160 families in pepper, similar to our study^23^. In addition, DE ncRNA targets were also found to be involved in the GA, ABA and IAA pathways. Further, numerous TFs, such as bHLH, NAC, MYB, bZIP, and WRKY, have also been reported to participate in the regulation of fruit ripening in tomato and pepper fruit^34,49^. Our study revealed that several lncRNAs and miRNA targets were the bHLH, TCP, bZIP, MYB and WRKY TFs and squamosa promoter-binding-like protein, which is an important part of the fruit ripening regulatory network.

CircRNAs were found to act as “miRNA sponges” and were found to be enriched with functional miRNA binding sites^54,55^. Several lncRNAs can form internal structures for certain biological outputs, such as sRNA production, as can primary miRNAs (pri-miRNAs); lncRNAs can also compete with miRNAs for binding sites on shared target mRNAs; furthermore, lncRNAs can bind to miRNAs to “communicate” with other RNA targets^56–59^. CircRNAs, lncRNAs and mRNAs can act as ceRNAs in gene regulation^9^. To date, there has been no report on the ncRNA networks and ceRNAs in bell pepper fruit. Here, we analyzed circRNA-mRNA-miRNA, miRNA-mRNA-lncRNA and circRNA-miRNA-mRNA-lncRNA networks and found that several vital enzymes and TFs were involved in the coordinated regulatory networks, such as *ACO1/4, ARF8, PSY1, WRKY, ERF039, lycopene epsilon cyclase, beta-galactosidase*, and *PG, pectin acetylesterase 9*, all of which are important regulators involved in fruit ripening^3,6,35,60^. In addition, the ceRNA networks were parsed, revealing that two important regulators, ethylene-responsive transcription factor RAP2-7 and eugenol synthase 1, may participate in the pepper fruit ripening process^35,61^. These findings provide a theoretical basis for deciphering novel mechanisms of fruit ripening and functionally characterizing ceRNA networks in future studies.

## Supplementary information

Appendix.1

Appendix.2

Appendix.3 and Appendix.7

Appendix.4

Appendix.5

Appendix.6
