# Supplementary material for: Network analysis of noncoding RNAs in pepper provides insights into fruit ripening control
Source: Sci Rep. 2019 Jun 19;9:8734. doi: 10.1038/s41598-019-45427-1 (PMC6584694; doi:10.1038/s41598-019-45427-1)
Supplement: Supplementary file 3 — Appendix.3 and Appendix.7 [file 41598_2019_45427_MOESM3_ESM.pdf]

Network analysis of non-coding RNAs in pepper provides insights into fruit ripening control

Jinhua Zuo[1,2*], Yunxiang Wang[3], Benzhong Zhu[4], Yunbo Luo[4], Qing Wang [1*], Lipu Gao[1*]

[1] Key Laboratory of Vegetable Postharvest Processing, Ministry of Agriculture, Beijing Key Laboratory of Fruits and Vegetable Storage and Processing, Key Laboratory of Biology and Genetic Improvement of Horticultural Crops (North China) of Ministry of Agriculture, Key Laboratory of Urban Agriculture (North) of Ministry of Agriculture, Beijing Vegetable Research Center, Beijing Academy of Agriculture and Forestry Sciences, Beijing 100097, China

[2] Boyce Thompson Institute for Plant Research, Cornell University Campus, Ithaca, NY, 14853, USA.

[3] Beijing Academy of Forestry and Pomology Sciences, Beijing Academy of Agriculture and Forestry Sciences, Beijing, 100093, China

[4] Laboratory of Postharvest Molecular Biology of Fruits and vegetables, Department of Food Biotechnology, College of Food Science and Nutritional Engineering, China Agricultural University, Beijing 100083, China

[*]Corresponding author, E-mail address: zuojinhua@126.com;

Appendix.3 The expressions of lncRNAs vs mRNAs

The expression of of lncRNAs vs mRNAs

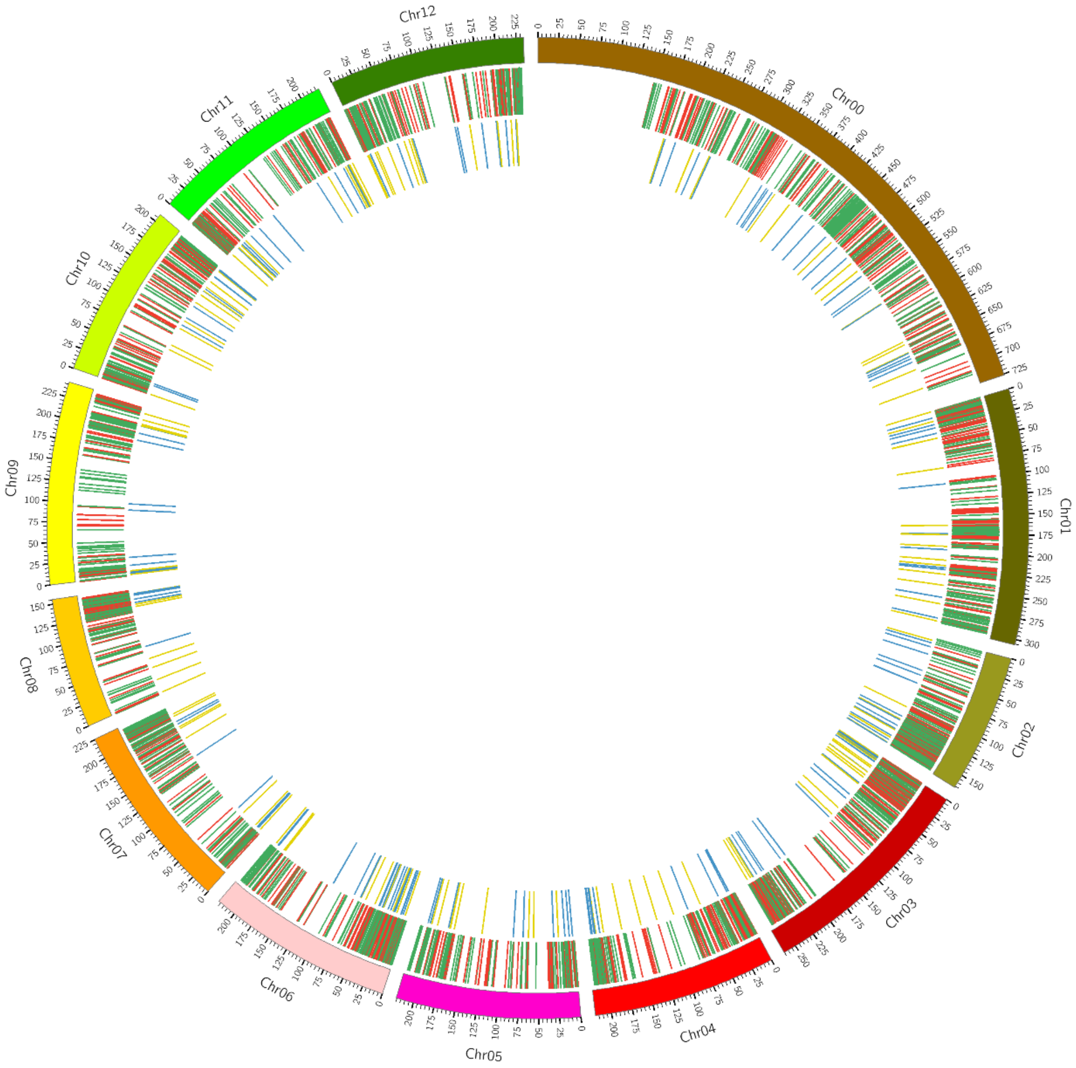

The Comparison of Isoform of lncRNAs and mRNAs

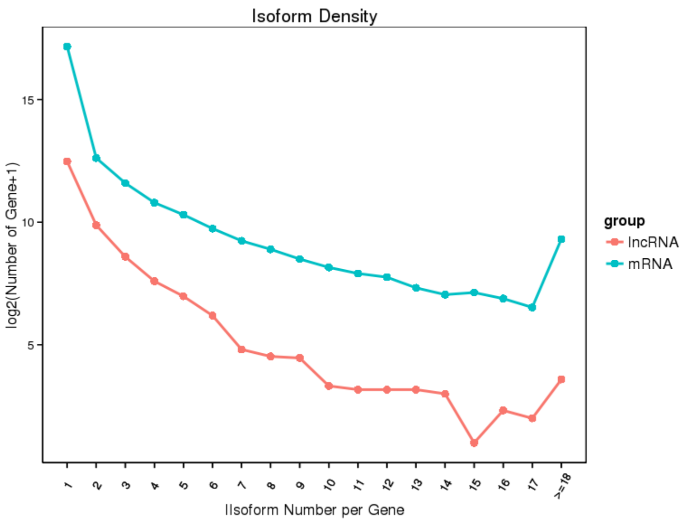

The interactive analysis of the lncRNAs and mRNAs

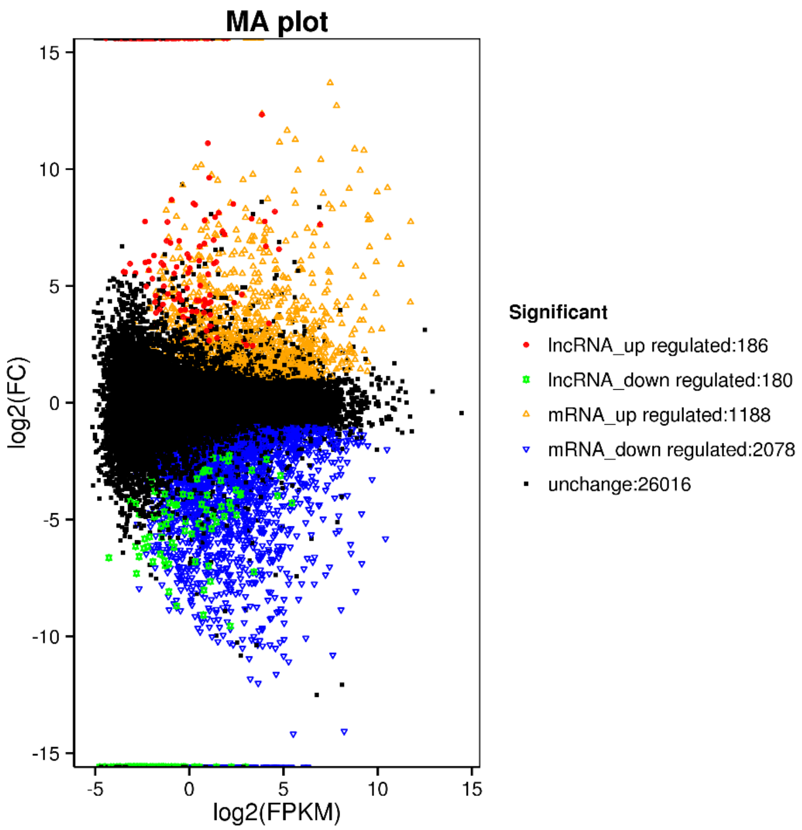

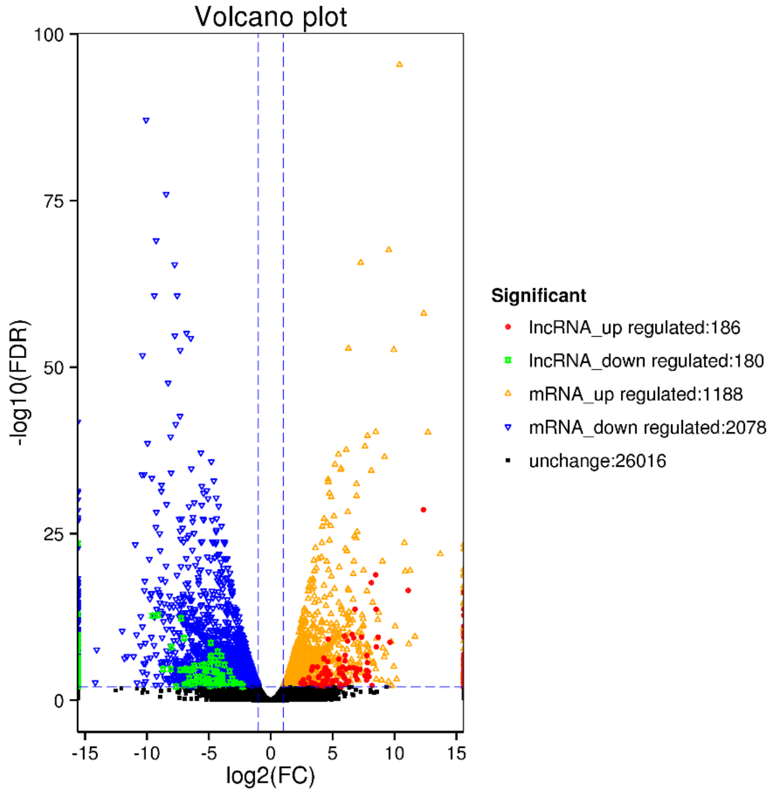

Appendix.7 The ncRNAs and mRNAs network related figures

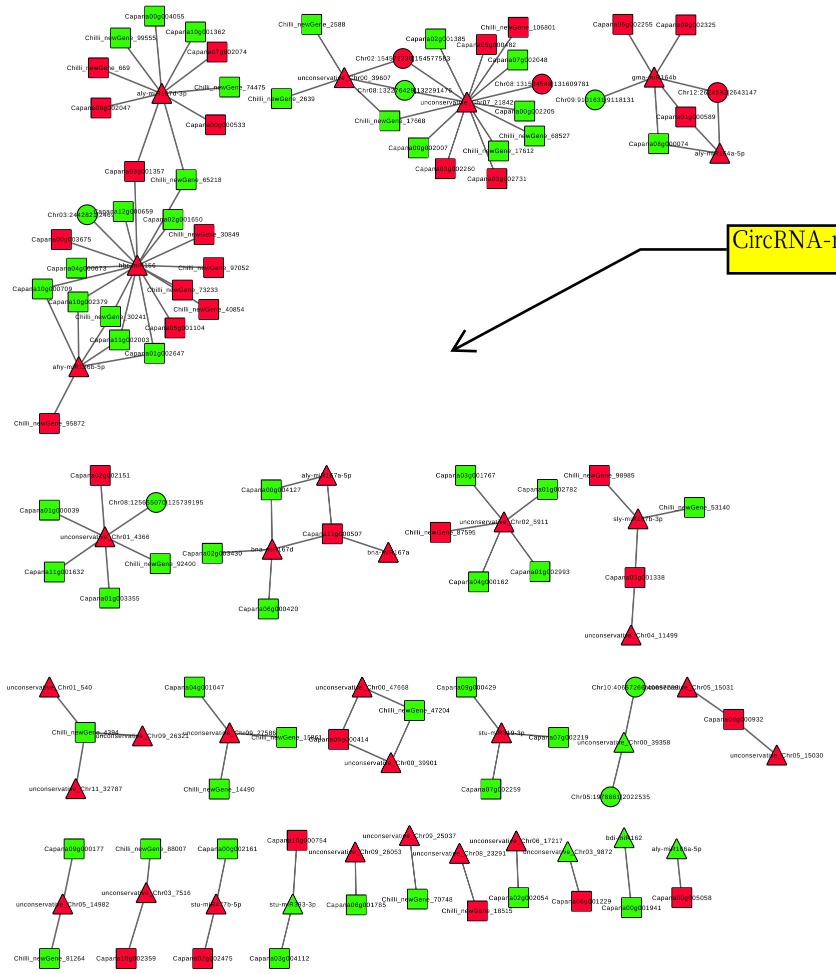

CircRNA-mRNA-miRNA-Network

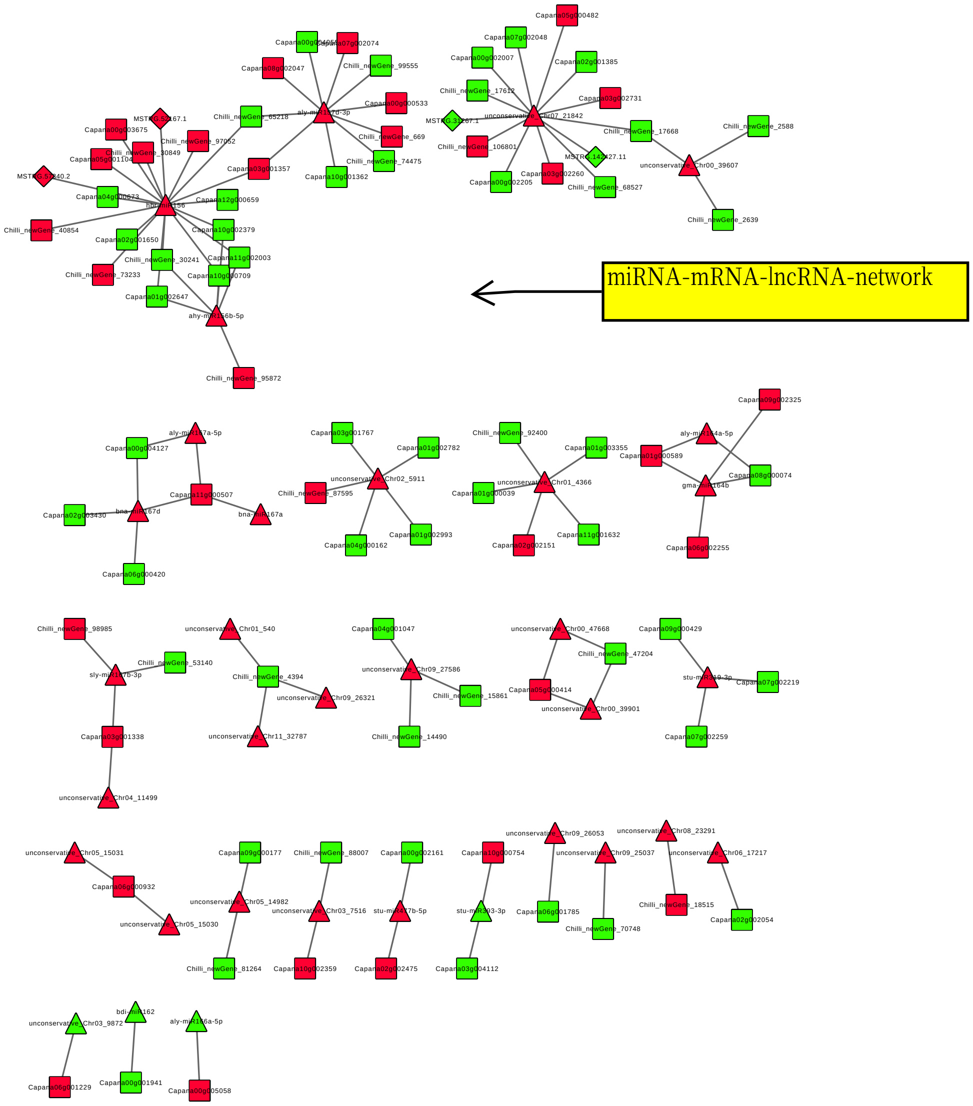

miRNA-mRNA-lncRNA-network
Chilli_newGene_65218
Chilli_newGene_99555
Chilli_newGene_669
Chilli_newGene_74475
Capana10g001362
Capana08g002047
Chilli_newGene_97052
Chilli_newGene_40854
Chilli_newGene_73233
Chilli_newGene_30241
Capana01g002647
Capana12g000659
Capana10g002379
Capana11g002003
Chilli_newGene_95872
Capana05g000482
Capana07g002048
Capana00g002007
Capana02g001385
Chilli_newGene_17612
Chilli_newGene_106801
Chilli_newGene_17668
Chilli_newGene_68527
Chilli_newGene_2588
Chilli_newGene_2639
Capana00g004127
Capana02g003430
Capana06g000420
Capana11g000507
Capana03g001767
Capana01g002782
Chilli_newGene_87595
Capana04g000162
Capana01g002993
Chilli_newGene_92400
Capana01g003355
Capana01g000039
Capana11g001632
Capana08g000074
Capana08g002325
Chilli_newGene_98985
Chilli_newGene_53140
Capana03g001338
Chilli_newGene_4394
Capana04g001047
Chilli_newGene_15861
Chilli_newGene_14490
Chilli_newGene_47204
Capana05g000414
Capana09g000429
Capana07g002219
Capana07g002259
Capana06g000932
Capana09g000177
Chilli_newGene_88007
Capana00g002161
Capana10g000754
Capana06g001785
Chilli_newGene_70748
Chilli_newGene_18515
Capana02g002054
Chilli_newGene_81264
Capana10g002359
Capana02g002475
Capana03g004112
Capana06g001229
Capana00g001941
Capana00g005058

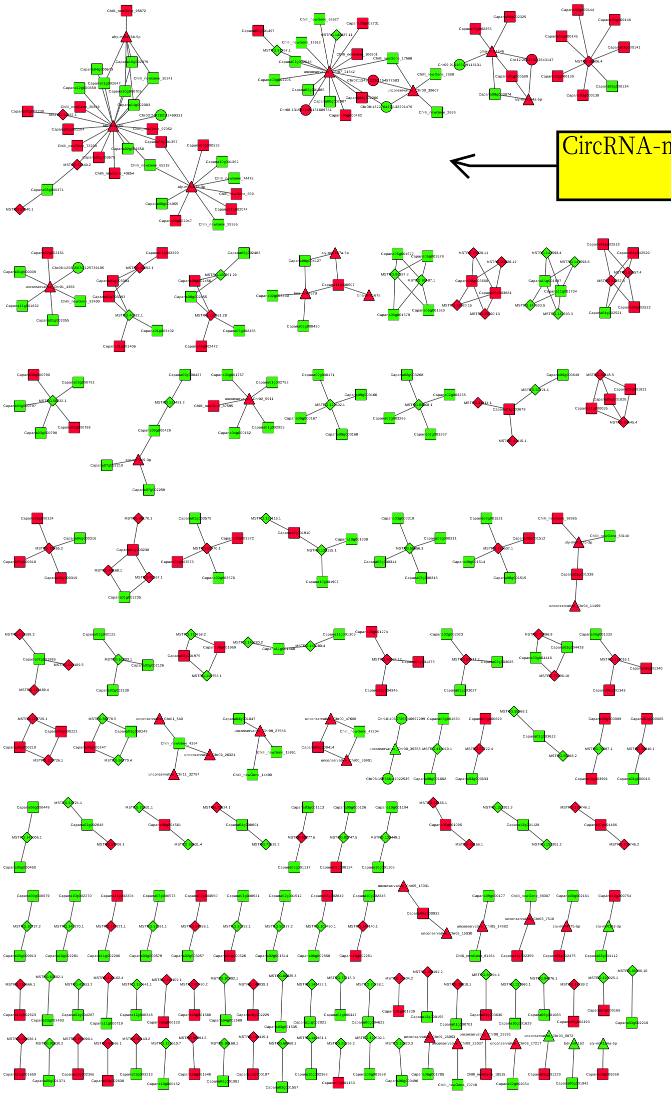

CircRNA-miRNA-mRNA-lncRNA-network

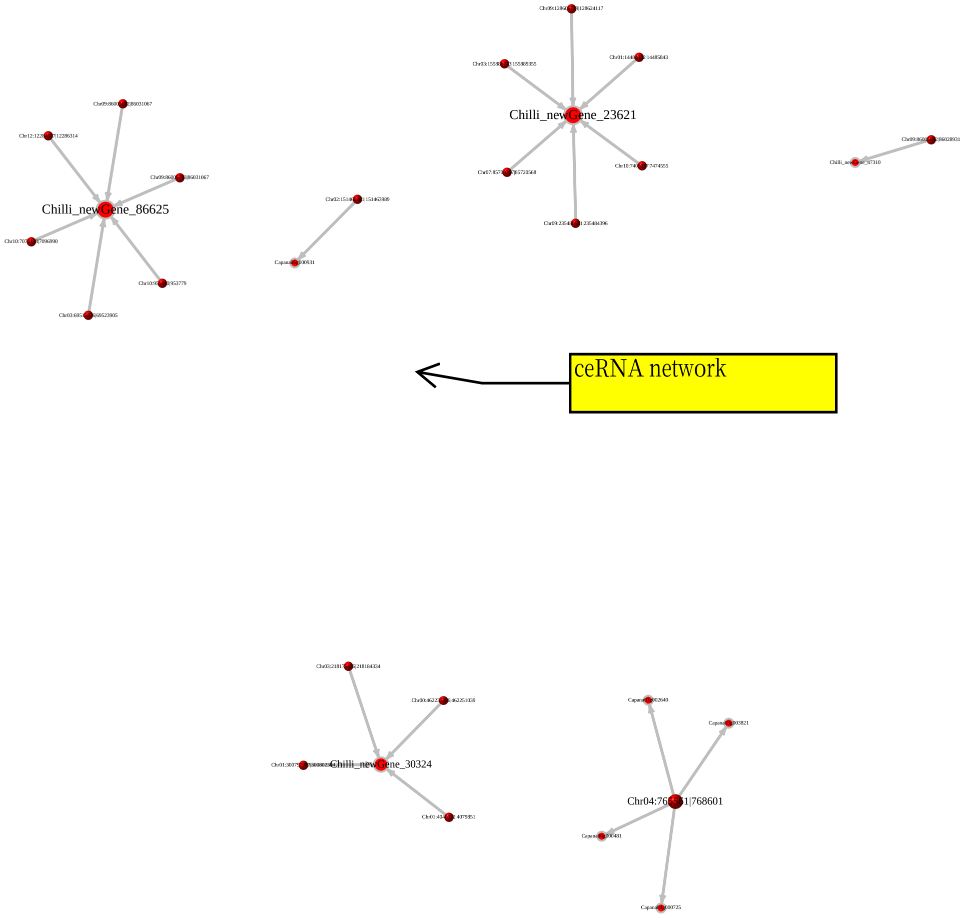

Chilli_newGene_86625
Chilli_newGene_23621
ceRNA network
Chilli_newGene_30324
